# Status of theileriosis among herbivores in Iran: A systematic review and meta-analysis

**DOI:** 10.14202/vetworld.2018.332-341

**Published:** 2018-03-19

**Authors:** Masoud Soosaraei, Mousa Motavalli Haghi, Fariborz Etemadifar, Mahdi Fakhar, Saeed Hosseini Teshnizi, Hajar Ziaei Hezarjaribi, Shabnam Asfaram

**Affiliations:** 1 Student Research Committee, Department of Parasitology, School of Medicine, Mazandaran University of Medical Sciences, Sari, Iran; 2 Student Research Committee, Department of Parasitology and Mycology, School of Medicine, Hamadan University of Medical Sciences, Hamadan, Iran; 3 Molecular and Cell Biology Research Center (MCBRC), Department of Parasitology, School of Medicine, Mazandaran University of Medical Sciences, Sari, Iran; 4 Biostatistician, Infectious and Tropical Diseases Research Center, Hormozgan University of Medical Sciences, Bandar Abbas, Iran

**Keywords:** epidemiology, Iran, livestock, systematic review, *Theileria* spp

## Abstract

**Aim:**

Theileriosis is a protozoal disease caused by *Theileria* spp. mostly in warm-blooded vertebrates worldwide. It is one of the common tick-borne diseases among domestic animals in tropical and sub-tropical regions, which have a variety of unlikable effects on health economy and animal welfare. In the present study, the prevalence of theileriosis among domestic farm animals in Iran was systematically evaluated.

**Methods:**

To identify the related papers, 10 English and Persian databases, including PubMed, Science Direct, Scopus, Web of Science, Medical Subject Headings, Google Scholar, Magiran, Barakatns (formerly Iranmedex), Elm net, and Scientific Information Database, were appraised for articles published throughout 1999-2017.

**Results:**

A total of 56 papers, providing the examination of 11,317 cattle, 9394 sheep, 2991 buffaloes, 1504 horses, 600 goats, and 212 donkeys were analyzed, matching for the prevalence of theileriosis from different parts of Iran were permitted for our allowing checklist. The overall prevalence of theileriosis among domestic herbivores was expected to be 19% (95% confidence interval: 15%, 22%). Our findings highlighted the average of the maximum prevalence in Razavi Khorasan (60.4%) and West Azerbaijan (49.1%) and the minimum in Mazandaran (1.1%) and East Azerbaijan provinces (2.2%), respectively. The high prevalence of *Theileria* infection in the herbivores (mainly sheep) verifies the well-known enzootic episode of theileriosis in Iran, predominantly in northeastern and western parts of the country.

**Conclusion:**

Our results suggested updated and imperative information on the true burden of theileriosis in Iran. Moreover, it could be supporting the gaps among monitoring, prevention, and control arrangements to improve the health economy, particularly among dairy farm animals.

## Introduction

Theileriosis is a tick-borne hemoprotozoal tropical disease in various warm-blooded vertebrates mainly domestic and wild mammals caused by protozoan parasites belonging to the *Theileria* spp. [1,2]. *Theileria* is a genus of protozoan that belongs to the phylum Apicomplexa (order: Piroplasmida and family: Theileriidae), which is transmitted by ixodid hard ticks acting as natural vectors. The parasite life cycle is well-known by extra-erythrocytic merogony in the lymphocytes and histiocytes, following by invasion of the red blood cells by the merozoites [3]. Although, *Theileria* parasite infect mainly a variety of both domestic and wild livestock, there is no proof that *Theileria* spp. are threats to human population [2,4,5].

Cattle and buffaloes can be infected by various species of *Theileria* and infections differ from subclinical (known as mild) to malignant (known as severe). Almost nine mild and malignant species of *Theileria* causing bovine theileriosis are including *Theileria mutans, Theileria buffeli*, *Theileria orientalis, Theileria velifera, Theileria taurotragi* and *Theileria parva* (as malignant bovine theileriosis; known as East Coast fever)*, Theileria annulata* (known as tropical bovine theileriosis)*, Theileria sergenti*, and *T. taurotragi*, respectively. Likewise, at least seven spices including *Theileria lestoquardi* (formerly *Theileria hirci*), *Theileria luwenshuni, Theileria uilenbergi* (causing malignant ovine theileriosis), and *Theileria separata*, *Theileria ovis, Theileria recondita* (causing mild ovine theileriosis*)*, and *T. annulata* are naturally causal agents of ovine theileriosis with worldwide distribution [6]; although recently, *T. annulata*, as causal agent of malignant bovine theileriosis, has been reported in southern Iran [7,8].

Laboratory diagnosis of theileriosis was performed mostly by detecting schizonts in Giemsa-stained thick and thin smears from blood or lymph node fine needle aspiration. Several conventional and novel diagnostic tools vary from low to high sensitivity, such as polymerase chain reaction (PCR) test, enzyme-linked immunosorbent assays (ELISAs), or an indirect fluorescent antibody test (IFAT), were used for determining prevalence and differentiating the *Theileria* spp. [6,9-11]. In spite of the presence of a variety of studies concerning theileriosis among livestock in Iran, there is inapplicable data about the true burden of it in these animals to forecast the financial burden and well-known capacity for control and prevention planning.

Concerning the influences of theileriosis on economy and animal welfare, more deliberations would be crucial for the epidemiological aspects and the approaches to screening panels in Iran. To the best of our knowledge, there is no systematic review and meta-analysis on the subject of animal theileriosis in Iran; thus, the main goal of our study was to find out the present status of ovine, bovine, and equine theileriosis in the country.

## Methods

### Ethical approval

Ethical approval is not needed for this kind of study.

### Searching approach

**Figure-1:**
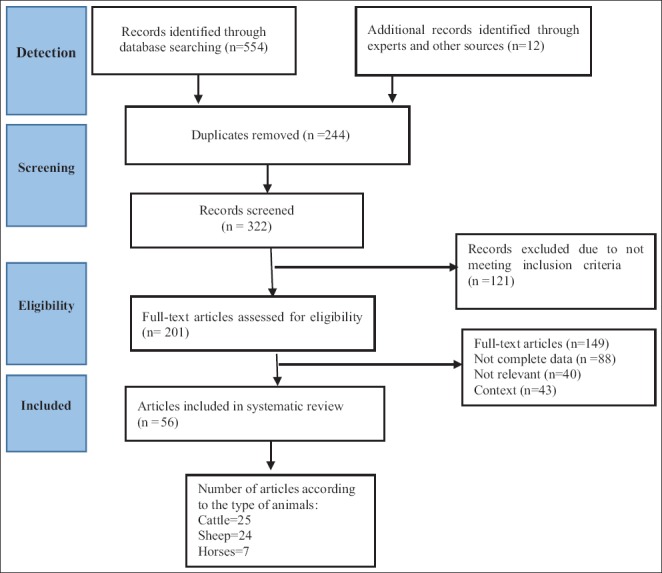
Flow diagram of classification of papers for inclusion in this systematic review and meta-analysis.

For searching purpose, 10 English and Persian databases, including PubMed, Science Direct, Scopus, Web of Science, Medical Subject Headings, Google Scholar, Magiran, Barakatkns (formerly Iranmedex), Elm net, and Scientific Information Database, were chosen during 1999-2017. To exploring the articles, the terms including: *Theileria*, *Theileriosis, T. orientalis, T. annulata, T. hirci, T. lestoquardi, T. ovis, Theileria equi*, cattle, buffalo, bovine, sheep, goat, ovine, caprine, horse, donkey, equine, and “Iran” alone or in combination were used. To avoid the risk of selection bias in this study, the inclusion criteria were clearly classified and studied. Experimental studies, clinical trials, duplicates, case reports, monkey, carnivores, camel, and studies out of Iran were expelled. All descriptive studies corresponding to the prevalence of ovine, bovine, and equine theileriosis were reviewed. The stages of the study plan are briefly explained in Figure-1.

### Data extraction

The data were extracted from the included studies by four reviewers (M. Soosaraei, M. Motavalli Haghi, F. Etemadifar, and Sh.Asfaram), who used a standard form. Any disagreement was resolved by discussion between the four reviewers. If consensus could not be reached, two reviewers were consulted (M. Fakhar and H. Ziaei Hezarjaribi). The kappa index showed an agreement of 89% between the fives reviewers. The standard form consisted of the following variables: First author; year of publication, year of study, type of animal, place of conducted survey (Province), sample size, number of positive, and diagnostic laboratory methods (Table-1) [7,12-65]. The quality of selected studies was assessed using the STROBE scale (score under 7.75 low quality, 7.76-15.5 moderate and 15.6-23.5 moderate to height, and upper 23.6 height quality).

**Table-1:** Baseline features of included studies.

Animal	No. of examined	No. of positive	Species	Laboratory method	Place (province)	References
Sheep	1000	92	*T. lestoquardi*	Microscopy	Fars	[12]
Buffalo	2700	82	*T. annulata*	Microscopy	Khouzestan	[13]
Cattle	372	216	*T. annulata*	Microscopy	Razavi Khorasan	[14]
Sheep	300	29	*T. lestoquardi*	Microscopy	Lorestan	[15]
Cattle	390	15	*Theileria*	Microscopy	Chaharmahal va Bakhtiari	[16]
Sheep	300	39	*Theileria*	Microscopy	Mazandaran	[17]
Cattle	124	61	*Theileria*	ELISA	West Azerbaijan	[18]
Sheep	200	7	*Theileria* spp.	Microscopy	Mazandaran	[19]
Sheep	840	100	*Theileria* spp.	Microscopy	South Khorasan	[20]
Cattle	600	34	*T. annulata*	Microscopy	Sistan and Baluchestan	[21]
Cattle	252	26	T. annulata	Microscopy	Sanandaj	[22]
Cattle	NR	NR	T. annulata	Semi-nested PCR	Tehran, Fars, Sistan, and Baluchestan	[23]
Sheep	150	71	*Theileria* spp., *T. ovis*, *T. lestoquardi*	PCR	Sistan and Baluchestan	[24]
Sheep	100	60	*Theileria* spp., *T. lestoquardi*	PCR	South Khorasan	[25]
Cattle	160	110	*Theileria* spp., *T. annulata*	Microscopy	Razavi Khorasan	[26]
Sheep	100	56	*T. ovis, T. lestoquardi*	PCR	East and South-East provinces	[27]
Sheep	470	21	*T. lestoquardi, T. ovis*	PCR	10 various regions of Iran	[28]
Sheep	220	181	*T. lestoquardi, T. ovis*	PCR and microscopy	5 various regions in eastern half of Iran	[24]
Cattle	160	16	*T. annulata and T. orientalis*	Semi-nested PCR	Golestan	[29]
Sheep	200	11	*T. ovis*	Microscopy	West Azerbaijan	[30]
Cattle	100	22	*T. annulata*	IFAT	West Azerbaijan	
Cattle	160	20	*T. annulata*	PCR	Golestan	[31]
Cattle			*T. annulata*	PCR-RFLP	Kurdistan and West-Azerbaijan	[32]
Cattle	200	63	*T. annulata*	Microscopy	Kerman	[33]
Sheep	250	101	*T. lestoquardi and T. ovis*	Nested PCR	Western half of Iran (Sari, Rasht, Urmia, Ilam, and Ahvaz)	[7]
Cattle	52	19	*T. annulata*	PCR-RFLP	Azerbaijan	[34]
Cattle	52	30	*T. annulata*	PCR-RFLP	Kurdistan and Kermanshah	
Cattle	1202	706	*T. annulata*	PCR	Isfahan, Khuzestan, Chaharmahal va Bakhtiari, Kohgiluyeh va Boyer Ahmad and Lorestan	[35]
Cattle	160	34	*T. orientalis* and *T. annulata*	Semi-nested PCR	Golestan	[36]
Sheep	165	9	*T. annulata* and *T. ovis*	PCR	Tehran	[37]
Sheep	568	73	*Theileria* spp.	Microscopy	Ilam	[38]
Sheep	100	12	*Theileria* spp.	Microscopy	Lorestan	[39]
Sheep	300	6	*Theileria* spp.	Microscopy	Tehran	[40]
Sheep	100	46	*T. ovis* and *T. annulata*	Semi-nested PCR	Fasrs	[41]
Sheep	90	68	*T. ovis* and *T. lestoquardi*	Semi-nested PCR	North Khorasan	[42]
Sheep	452	295	*T. ovis*, *T. lestoquardi* and *T. annulata*	Semi-nested PCR	Razavi Khorasan	[43]
Cattle	176	42	*Theileria* spp.	PCR	Isfahan	[44]
Cattle	270	20	*T. annulata*	PCR	Yazd, North Khorasan and Mazandaran	[45]
Horses	165	47	*T. equi*	PCR	Khuzestan	[46]
Horses	240	41	*T. equi*	PCR	West Azerbaijan	[47]{Malekifard, 2014 #2067}{Malekifard, 2014 #2067;Malekifard, 2014 #2067}
Horses	100	53	*T. equi*	IFAT	North Khorasan	[48]
Horses	205	45	*T. equi*	PCR	North Khorasan and Yazd	[49]
Sheep	119	106	*Theileria* spp.	PCR	Khuzestan	[50]
Cattle	150	43	*Theileria* spp.	PCR	Isfahan	[51]
Donkeys	106	54	*T. equi*	Multiplex-PCR	North Khorasan	[52]
Cattle	138	13	*T. annulata*	PCR	Kermanshah	[53]
Horses	59	21	*T. equi*	PCR	Khuzestan	[54]
Sheep	150	19	*T. lestoquardi* and *T. ovis*	PCR	Lorestan	[55]
Cattle	150	84	*T. annulata*	PCR	Kerman	[56]
Cattle	100	4	*T. annulata*	PCR	Yazd	[57]
Goats	100	0	*Theileria*	Microscopy	Razavi Khorasan	[58]
Sheep	80	59	*Theileria*	PCR	Sistan and Baluchestan	[59]
Cattle	160	60	*T. annulata*	PCR	Sistan and Baluchestan	[60]
Cattle	138	37	*Theileria*	Semi-nested PCR	West Azerbaijan	[61]
Goat	400	39	*T. lestoquardi*	PCR	West Azerbaijan	[62]
Cattle	193	76	*T. annulata* and *T. orientalis*	Molecular assays	West Azerbaijan	[63]
Buffalo	291	10				
Cattle	51	2	*T. lestoquardi* and *T. annulata*	PCR	Khuzestan	[64]
Horse	90	10	*T. equi*	PCR	Isfahan and Shahrekord	[65]

NR: Not reported, *T. lestoquardi=Theileria lestoquardi, T. annulata=Theileria annulata, T. ovis=Theileria ovis, T. orientalis=Theileria orientalis, T. equi=Theileria equi*, PCR=Polymerase chain reaction, IFAT=Indirect fluorescent antibody test, ELISA=Enzyme-linked immunosorbent assays, RFLP=Restriction fragment length polymorphism

### Effect measures

The outcome was the prevalence of *Theileria*, and this was obtained for each study by dividing the number of positive cases by the total sample size.

### Statistical analysis

The prevalence of each study was collected, and according to the binomial distribution, standard error 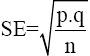 for each study was calculated and the inverse of SE each study considered as the weight of that study. The effect size (ES) for each study and pooled outcome revealed as a forest plot (reported as ES with a 95% confidence interval [95% CI]). Cochran’s heterogeneity statistics based on Chi-square test Q-test (p<0.1 as heterogeneities) and the I^2^ statistic which describes the percentage of variation across studies (values of 25%, 50%, and 75% indicate low, moderate, and high degrees of heterogeneity, respectively). At present heterogeneity, random effects model (Der Simonian Laird model) and otherwise applied fixed-effect model (Mantel Haenszel) were used to compute overall ES. Subgroup analyses were performed to investigate potential sources of heterogeneity from different sex and age. Egger’s tests were used to evaluate publication bias. All statistical analyses were fulfilled with the statistical software package (Stata) version 11.1. (Stata Corp LP, College Station, TX, USA). p<0.05 was measured statistically significant.

## Results

Among each one of the databases investigated during 19 years from 1999 to 2017, overall 56 articles were fitting to be integrated into our systematic review and meta-analysis. All papers were assigned and assessed the prevalence of theileriosis among herbivores including sheep, goat, cattle, buffalo, horse, and donkey in Iran. Absolutely, 11,317 cattle, 9394 sheep, 2991 buffaloes, 1504 horses, 600 goats, and 212 donkeys were analyzed, respectively. In general, because of the restricted data fulfilled on camel theileriosis in Iran, we included just articles correlated to the disease among domesticated herbivores except for camel.

**Figure-2:**
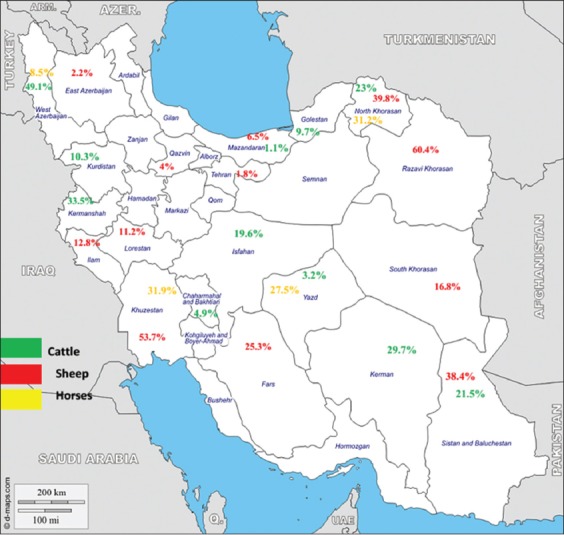
Detailed presentation of overall distribution of *Theileria* spp. infection in the provinces of Iran.

**Figure-3:**
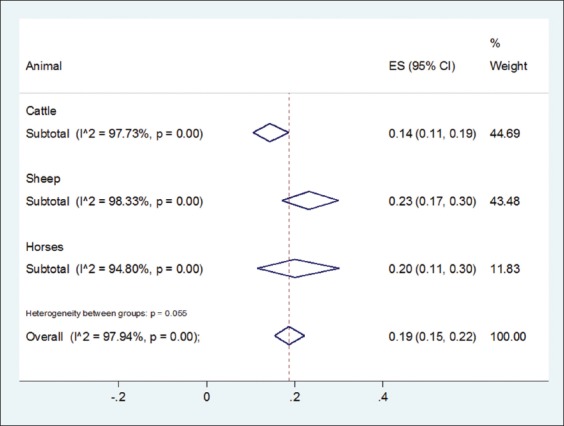
Estimate of the prevalence of *Theileria* infection among herbivores based on 56 studies in different years and areas in Iran. The pooled random effect size and 95% confidence interval represents by diamond, 19% (15-22%). Overall heterogeneity based on random effect model was showed by I^2 (^97.94%, p<0.001). The vertical dash line represents overall estimate, and the vertical solid line represents the value of null hypothesis.

The mean of scores for the STROBE scale was to be found 21.73 which performed quality of these studies was moderate to height. As indicated by a random effect meta-analysis (I^2^=98.94%, p<0.001) the pooled event of *Theileria* infection in Iran was acquired 19% (95% CI: 15%, 22%). The prevalence rate of ovine (sheep and goats), bovine (cattle and buffaloes), and equine (horses and donkeys); theileriosis was 23.0% (17.0-30.0%), 14.0% (11.0-19.0%), and 20.0% (11.0-30.0%), respectively; a significant statistically difference was observed among them (z=5.80, df=2, p=0.05) (Figures-2 and 3).

Besides, our results showed the prevalence of *Theileria* infection among sheep (23%) which was considerably too much than other herbivores (p<0.001); and prevalence of *T. ovis* and *T. annulata* in sheep and cattle was significantly higher than other ones, respectively (p<0.001).

In addition, the highest prevalence of *T*. *equi* infection in horses 19.0% (11.0-29.0%) was detected by IFAT (51%) and the lowest by microscopic methods (7%). There was significant difference between prevalence of laboratory methods which used for diagnosing equine theileriosis for horses (Q-test=137.25, df=2, p<0.001).

The subgroup analysis showed PCR method had the most prevalence for sheep, 33.0% (95% CI: 20.0-48.0%) and a significant difference between prevalence of *Theileria* infection and laboratory techniques used among sheep (Q-test=9.5, df=3, p=0.02). Moreover, our meta-analysis showed a significant difference among *Theileria* species in sheep (Q-test=41.2, df=3, p<0.001) and cattle (Q-test=180.43, df=4, p<0.001), correspondingly. In addition, the analysis confirmed a significant difference between prevalence of *Theileria* infection and laboratory methods used for cattle (Q-test=699.32, df=5, p<0.001).

The results of Egger’s test for each of animal subgroups provided no evidence of publication bias in the studies (p>0.1) (Table-2). Therefore, it appears that both studies with low and high prevalence were contributed in this meta-analysis. The consequences of subgroup examination independently for type of laboratory methods and *Theileria* spp. infection showed that these two elements are two major sources of heterogeneity among prevalence of theileriosis for studies related to cattle and sheep, except horses.

**Table-2:** The results of Egger’s test to assess publication bias.

Animals	Number of studies	bias	p
Cattle	25	1.08	0.21
Sheep	24	1.37	0.56
Horses	7	2.30	0.11
Overall	56	1.67	0.13

## Discussion

Our study is the first systematic review on theileriosis in Iran. Theileriosis is an economically important protozoal disease among domesticate livestock in Iran with overall 19% infection rate, and the infection rates in sheep are 23%, which cause terrible impacts on the economy. The disease is widespread and well known in several parts of Iran, which affects animals’ husbandry and their productions in the country. The majority cases of theileriosis occurred in June-July and the lowest in March-April in northwestern and southeastern Iran [4,66]. In addition, the high prevalence of ovine theileriosis in different areas of Iran is probably identified with a few issues, for example, vector seasonal frequency, weather and environmental alterations, host susceptibility, ticks resistance to insecticide, high frequency of tick-infested in sheep versus cattle, and insufficient preventive policies. [4,67-70].

Several hard tick genera including *Amblyomma*, *Haemaphysalis*, *Hyalomma*, and *Rhipicephalus* as main vectors of theileriosis are scattered in all ruminant grazing lands, and accordingly, the chance of transmission of it is increased [48].

In our study, the pooled prevalence of livestock theileriosis is approximated to be 19% in Iran. Moreover, the prevalence rate of theileriosis in various geographical regions demonstrated that there were assorted zones in Iran with high prevalence rates in cattle with 58% in Razavi Khorasan, northeastern, and 57.6% in Kermanshah, western Iran, in sheep with 71% in Sistan and Baluchestan, Eastern, 70%, 55.6% in North Khorasan and Razavi Khorasan, respectively; in horses with 53% in North Khorasan provinces. Furthermore, the average prevalence of infection rate was notably lower in the central and northern provinces of the country.

Our findings are in symmetry with a study carried out on sheep and goats in Pakistan, eastern neighborhood of Iran, in 2012, by microscopic examination, 11.2% samples were positive for *Theileria* spp. The prevalence of *Theileria* spp. was 13.9 % and 8.2% in sheep and goat, respectively [71].

In Turkey, as western neighborhood of Iran, the prevalence of *T. buffeli/T. orientalis* was reported between 0.9% and 13.6% using PCR, and *T*. *annulata* was reported between 0% and 60.5% using microscopic method. The seroprevalence of *T. annulata* was found between 1.8% and 91.4% by IFAT. The prevalence of *T*. *annulata* by molecular techniques was between 15.4% and 61.2%. The prevalence of *T. recondita/T. ovis* by microscopic examination of thin blood smears was varied from 0% to 41.3% and its seroprevalence was found to be between 8.2% and 63.2% by IFAT. Weather conditions, the tick seasonal activities, and ecological conditions of Turkey and Kurdistan region of Iraq are extremely close to western and northwestern Iranian provinces, e.g., West and East Azerbaijan [32,72-75].

On the whole, our study showed that the average prevalence rate of ovine, bovine, and equine theileriosis was 23, 14, and 20%, respectively. In this study, five species of *Theileria* were identified which abundantly found in different livestock, would be considered as causal agents of theileriosis in the country. Our data showed that ovine theileriosis caused by *T. lestoquardi* infection is common in southern, southwestern, and southeastern parts of Iran and *T. ovis* is prevalent all over the country; even though the latest species is the main species in northern, western, and northwestern areas of Iran [7,24,43].

Regarding bovine theileriosis in Iran, our data are similar to the prior epidemiological surveys of *T. orientalis* and *T. annulata* in neighboring countries. For example, in four districts of Punjab, Pakistan, the pooled prevalence of *T. orientalis* was approximately 24.5, 6, and 6.1% by multiplexed tandem PCR method in the imported and native Pakistani cattle and buffaloes, respectively [76].

Equine theileriosis is caused due to *T. equi* and *Babesia caballi* and it is widespread in the most of tropical parts of countries in Europe, Africa, Asia, and countries surrounding Iran such as Turkey [77,78], Oman [79], Saudi Arabia [80], Kuwait [81], United Arab Emirates [82], and Iraq [83] as well as from other countries, found varying degrees of parasite prevalence. Overall, *T. equi* is most common and malignant than *T. caballi* in many endemic parts of the world including Iran [49].

In a study from Iran, with PCR assay showed infection with *T. equi* in 50.9% of the donkeys [52]. The frequency of *T. equi* infections was recorded from 31.8% of donkeys in Brazil using PCR method [84], 0.5-12% of blood smears of donkeys in Ethiopia [85-89]. Seropositivity rates of *T. equi* infection were in73.8% of donkeys in Brazil [84], 55.7% in Ethiopia [87], 9.6% in China [88], 47.2% in Spain [89], and 4-13% in Turkey [90,91].

Our findings and recent data show that *T. equi* and *T. caballi* were common in horse populations in provinces of Iran including East and West Azerbaijan, North Khorasan, and Khuzestan [23,47].

Herbivores theileriosis is answerable for high mortality and morbidity of livestock and subsequently fizzling economy, stunning effects on traditional and industrial animal breeding, in addition falling control approaches [4,92].

The diagnosis of theileriosis is based on traditional methods including microscopic examination (mostly thick and thin Giemsa-stained blood smears), and clinical symptoms. On the other hand, since the microscopic method has low sensitivity, this technique is not being reliable for the detection of asymptomatic and or subclinical infections because of the low parasitemia and or low virulence [6,93]. Moreover, serological assays such as ELISA and IFAT have also many disadvantages such as cross-reactivity between various species. Recently, molecular tools have picked up notoriety and prominence for detection and characterization proof of various pathogens. Altogether, serological and molecular tools may be appropriate in mild and also asymptomatic and or subclinical infections [6,93-95].

Given that there is a large number of studies about distribution status of ruminant theileriosis contrasting babesiosis [62] and a limited number of studies regarding equine theileriosis in Iran. Moreover, the most of studies are concentrated on ovine theileriosis, so an urgent need for updated data about the prevalence of theileriosis in Iranian equine theileriosis would be required. Nonetheless, a few unpublished data concerning ovine and bovine theileriosis are available in local provincial veterinary health and management centers gathered from private veterinary clinics and diagnostic laboratories in the various parts of Iran, which even though they are not exactly noteworthy and valid for appraisal.

## Conclusion

The high occurrence of *Theileria* infection in domestic livestock in Iran, frequently among sheep, confirm the endemic and stable situations of theileriosis in the studied regions particularly northeastern and western provinces of the country and maybe a warning for animal welfare and health economy. In brief, our data offer valuable and encouraging information as regards the current situation of theileriosis in domestic herbivores in Iran, which might be useful for active and passive surveillance and preventing plans for the disease. Further investigation and monitoring will be needed to expand the surveillance and control policies, such as quite vaccination full coverage and improvement the traditional diagnostic tools and assessment the pesticide resistance in ticks to reduce the mortality and morbidity of theileriosis among livestock and consequently decrease the risk of outbreaks and economic failure and public health hazardous in Iran.

## Authors’ Contributions

MS, MMH, FE, and SA made searching strategy paper selection and wrote the manuscript draft, MF and HZH designed all steps of the study, SHT analyzed the extracted data. All authors read, revised, and approved the final manuscript.
